# Examining Cesarean Section Rates in Ghana's 10 Regions Over a Decade a Comprehensive National Investigation

**DOI:** 10.1155/2024/3774435

**Published:** 2024-11-09

**Authors:** Senyefia Bosson-Amedenu, Abdulzeid Anafo, Ahmed Ouerfelli, Nabil Ouerfelli, Noureddine Ouerfelli

**Affiliations:** ^1^Department of Mathematics, Statistics and Actuarial Science, Takoradi Technical University, Takoradi, Western Region, Ghana; ^2^Department of Mathematical Science, University of Mines and Technology, Tarkwa, Western Region, Ghana; ^3^Department of Mathematics, Faculty of Sciences of Bizerte, University of Carthage, Zarzouna, Bizerte, Tunisia; ^4^Regional Department of Health Services, Regional Directorate of Public Health of Zaghouan, Cité Menzah, Zaghouan, Tunisia; ^5^Laboratoire de Biophysique et Technologies Médicales, Université de Tunis El Manar, Tunis, El Manar, Tunisia

**Keywords:** cesarean section, healthcare planning, maternal health, quasilinearity, regional disparities

## Abstract

This study examines cesarean section (C-section) deliveries in Ghana from 2008 to 2017 in 10 regions, distinguishing between scheduled and emergency procedures. Scheduled C-sections target specific maternal conditions, such as advanced age, multiparity, and medical history, while emergency C-sections address acute fetal distress, preeclampsia, bleeding, and other urgent situations. The analysis reveals various regional patterns, with the Brong-Ahafo Region showing a potential deceleration after 2017 and the Upper West Region indicating a possible acceleration. The high number of C-sections in Greater Accra and Ashanti may be related to population density and health facilities. The study proposes empirical models, including linear, quadratic, and exponential components, emphasizing quasilinearity. The exponential model suggests transient and permanent phases of cesarean frequency, with the latter dominated by quasilinearity. Optimal parameter values are determined, which highlights the stability of the model. However, caution is advised when projecting too far into the future due to the inevitable slowing of observed trends. The findings offer insights for healthcare planning, resource allocation, and policymaking, emphasizing the need for region-specific approaches and ongoing monitoring of cesarean dynamics to inform nuanced interventions.

## 1. Introduction

A cesarean section (C-section) is a surgical method used for the delivery of a baby by making incisions in the abdomen and uterus of the mother. Although it is a common and often lifesaving procedure, understanding the various conditions and situations that require a cesarean delivery is imperative [1]. Studies on cesarean birth disparities in Ghana reveal significant challenges in access to this lifesaving procedure. Research indicates that the C-section rate in Ghana is notably lower than the global average, with rates reported at around 11% in 2016, as highlighted by the World Health Organization [2]. Disparities in C-section rates are particularly evident in rural areas, where access to healthcare facilities and skilled birth attendants is limited [3]. Additionally, maternal mortality rates associated with cesarean deliveries in Ghana are higher compared to those in developed countries, reflecting challenges in providing timely interventions during childbirth complications [4]. Sub-Saharan Africa as a whole also faces similar challenges. Studies show that C-section rates in sub-Saharan Africa are among the lowest globally, with many countries reporting rates below the WHO-recommended threshold of 10%–15% [5]. This suggests a substantial gap in access to essential obstetric care in the region, contributing to adverse maternal and neonatal outcomes. Efforts to address cesarean birth disparities in Ghana and sub-Saharan Africa include initiatives aimed at strengthening healthcare infrastructure, improving access to skilled birth attendants, and raising awareness about the importance of timely interventions during childbirth [6]. The maternal mortality rate for cesarean deliveries in the United States is approximately 2.2 per 100,000, which, while relatively low, is considerably higher than that associated with vaginal deliveries. For vaginal births, the maternal mortality rate is approximately 0.2 per 100,000. There are several reasons why it may not be advisable or possible for a fetus to be delivered vaginally. Some of these indications are rigid, as a vaginal birth could pose risks in specific clinical situations. For example, opting for cesarean delivery is often recommended if the patient has a previous classical cesarean scar or has experienced a uterine rupture in the past. However, given the potential complications associated with cesarean deliveries, extensive research has been conducted to explore ways to minimize the rate of cesarean procedures. Efforts have focused on reducing the occurrence of first-time cesareans, as many women who undergo an initial cesarean delivery are likely to also have subsequent children delivered by cesareans [1, 7–11]. Scheduled prophylactic C-sections are preplanned based on specific maternal and fetal considerations. Advanced maternal age (35 years), multiparous women (four times), pregnancies induced after sterilization, and maternal medical history (risk factors) are all factors that may necessitate a scheduled C-section. Elevated maternal age is linked to a higher risk of pregnancy complications, leading to the precautionary measure of cesarean delivery. Similarly, women with multiple previous pregnancies or those who have undergone sterilization may face increased risks, prompting the recommendation for a scheduled C-section. Additionally, specific maternal medical conditions or a history of complications in previous pregnancies may also require a scheduled cesarean delivery to ensure the safety of both the mother and the baby. Women who experience extreme fear of labor pain (algophobia) may opt for an emergency C-section to avoid the anticipated pain associated with vaginal delivery [12–16].

In 2017, the Ghana Health Service (GHS) reported that the national average for C-sections was approximately 16%. The Greater Accra Region had the highest rate at 24.3%, while the Upper East region had the lowest at 7.2%. The WHO has expressed concerns about the possible negative effects of C-sections, both immediately and in the long term. Consequently, the WHO suggested that the C-section rate for any population or country should ideally remain below 15%. Understanding the risk factors that contribute to these rates is considered crucial. A study by Banchani and Tenkorang [17] investigated risk factors for C-sections in Ghana using data from the Ghana Maternal Health Survey 2017. They found that despite 87% of Ghanaian women preferring vaginal delivery, C-section rates were increasing. Visser et al. [18] discussed the global rise in cesarean deliveries, emphasizing the need for better informed choices between vaginal and cesarean delivery. Alhassan [19] examined the prevalence and socioeconomic factors influencing C-section delivery in Ghana using data from the Ghana Multiple Indicator Survey 2017/2018, revealing a high prevalence and increasing practice of cesarean deliveries. Okyere et al. [20] explored inequalities in C-section prevalence in Ghana from 1998 to 2014, finding a significant increase in cesarean births during this period. In Ghana, extensive research has been conducted on C-sections. Although most of the studies have focused on socioeconomic, prevalence, and risks, few have investigated the trend of C-section rate by region in the Ghanaian population. More specifically, little attention has been paid to the regional trend of the C-section between the years 2008 and 2017. The purpose of the current study is to investigate the trend of the C-section in Ghana by comparing the trends by region at the population level.

## 2. Empirical Models and Methodology

### 2.1. Study Design

This research employs a retrospective cohort study design to comprehensively examine the patterns of cesarean births in Ghana from 2008 to 2017.

### 2.2. Sample Size and Sampling Technique

The study utilizes a large sample size of 692,045 cesarean births, obtained through a systematic sampling technique from hospital records across all 10 regions of Ghana from the national health database (Ghana's Ministry of Health).

The sample for this study was determined using hospital records from the national database maintained by Ghana's Ministry of Health. These records documented all C-section deliveries that took place across the 10 regions of Ghana between 2008 and 2017. The sample includes both scheduled and emergency C-sections recorded within this period.

A systematic sampling technique was used to ensure that the sample was representative of C-sections performed across the country. This technique involved selecting all available records of C-sections from registered hospitals in each region, ensuring full coverage of the period under investigation.

In total, the dataset comprises 692,045 C-section births, ensuring that every region's contribution to the overall C-section count was represented proportionally, reflecting the actual healthcare conditions and C-section rates in each region during the 10-year period. The large sample size allowed for a comprehensive analysis of both regional trends and national patterns.

### 2.3. Sample Distribution Among Regions

The distribution of the sample across the regions is proportional to the total number of C-sections recorded in each region from 2008 to 2017. The sample sharing among the regions is as follows:

• Greater Accra: 184,307
• Ashanti: 108,270
• Eastern: 76,105
• Brong-Ahafo: 74,002
• Central: 66,676
• Western: 65,090
• Volta: 49,938
• Northern: 37,231
• Upper East: 17,739
• Upper West: 12,687

This distribution reflects regional disparities in C-section delivery rates, which are influenced by factors such as population density, healthcare infrastructure, and socioeconomic conditions.

### 2.4. Eligibility Criteria

All cesarean births recorded in Ghanaian hospitals from 2008 to 2017 were eligible for inclusion in the study.

### 2.5. Exclusion Criteria

The study excluded the following cases:

• Vaginal births and nonsurgical deliveries.
• Pregnancies with incomplete or missing data in the hospital records.
• C-sections performed outside the formal healthcare system or unregistered in the national database.
• Multiple births where a combination of vaginal and cesarean deliveries was recorded (e.g., one twin delivered vaginally and the other via C-section).

### 2.6. Ethics Committee Approval

Ethics approval for this study was obtained from Ghana's Ministry of Health prior to data collection, ensuring adherence to ethical guidelines for the research.

### 2.7. Methodology Explanation

A retrospective cohort study design was employed, analyzing hospital records of cesarean births across Ghana's 10 regions from 2008 to 2017. A combination of scatter-point analysis, mathematical modeling (including linear, quadratic, and exponential models), and Fourier analysis was used to identify trends and fluctuations in cesarean birth frequencies. The least squares method was applied to optimize parameter values for the models. These methods allowed for the identification of both transient and permanent phases in the trends, providing a robust framework for understanding regional variations in C-section deliveries.

### 2.8. Data Analysis

#### 2.8.1. Scatter-Point Analysis

Scatter-point patterns in cesarean birth data were analyzed to identify potential trends, considering regional characteristics and variations.

### 2.9. Mathematical Modeling

Various mathematical models, including exponential, linear, second-degree polynomial, and third-degree polynomial, were proposed to capture fluctuations in cesarean birth frequencies. Correlation coefficients of these models were compared to select the most appropriate ones for analysis.

### 2.10. Optimization

The least squares method was employed to determine optimal parameter values for the selected mathematical models, enhancing the accuracy of trend predictions. Fourier analysis was employed to identify and analyze periodic variations in the relative deviation to linearity of cesarian births. The importance of Fourier analysis lies in uncovering cyclic patterns, particularly in time-series data. In the context of this research, Fourier analysis provided insights into the annual fluctuations in cesarian birth frequencies. Both methods collectively contributed to a robust analysis of the temporal evolution of cesarian births in Ghana.

## 3. Results and Discussions

### 3.1. First Analysis by Region

**Figure 1:**
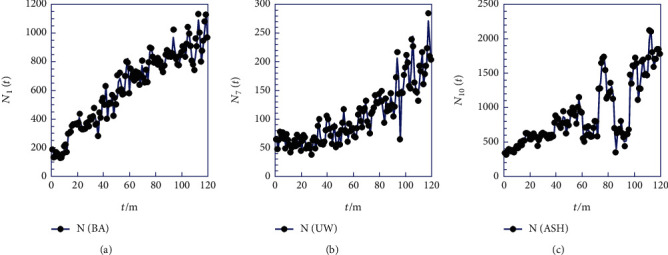
Frequency of cesarian births (*N*_i_) recorded for each month from 2008 to 2017 for three regions (Supporting Information) in Ghana. (a) Brong-Ahafo (BA); (b) Upper West (UW); (c) Ashanti (ASH).

In the initial phase, we examined the monthly occurrence of cesarean births (*N*_*i*_) from 2008 to 2017 for 10 regions (*i* = 1 to *i* = 10) in Ghana (Table 1). The graphical representation of (*N*_*i*_) against time (*t*) reveals varied scatter point patterns, indicating various behaviors. Some trends exhibit a globally concave shape, while others show a convex shape, etc.  Figure 1 illustrates this for three regions, namely, Brong-Ahafo (BA), Upper West (UW), and Ashanti (ASH).

Table 1 also provides an assessment of the total documented cesarean births (Ni, tot) for each region in Ghana from 2008 to 2017 (Equation (1)).

From Equation (1), we can calculate the total number of cesarian births (*N*_tot_) recorded in Ghana during the 10-year period from 2008 to 2017 (Equation (2)).

(1) $$\begin{array}{c} \;N_{i,\text{tot}}=\overset{t=t_{f}}{\underset{t=1}{\sum }}N_{i}\left(t\right) \end{array}$$

where *t*_*f*_ represents the final time (*t*_*f*_ = 120 months). From Equation (1), we can calculate the total number of cesarian births (*N*_*tot*_) recorded in Ghana during the 10-year period from 2008 to 2017 (Equation (2)).

(2) $$\begin{array}{c} N_{\text{tot}}=\overset{i=10}{\underset{i=1}{\sum }}N_{i,\text{tot}}=692045 \end{array}$$

Equation (3) represents the proportion of cesarean births relative to the median population. It gives an overall percentage of cesarean births for the entire country over the specified 10-year period.

(3) $$\begin{array}{c} N_{\text{rel}}=\frac{N_{\text{tot}}}{P}=\frac{692045}{27192180}=2.545\% \end{array}$$

The relative cesarean births, considering only the female population, amount to approximately 5.1%. This additional calculation provides a more specific percentage related to cesarean births in relation to the female demographic.

In summary, these equations and calculations provide a comprehensive assessment of cesarean births in Ghana, including the total number, relative proportions, and specific considerations related to the female population.

Upon initial examination, Figure 1(a) presents a point distribution with an apparent convex shape in the Brong-Ahafo region, hinting at a potential deceleration in the phenomenon beyond 2017. On the contrary, Figure 1(b) illustrates an upward trend with a concave appearance in the Upper West Region, suggesting a possible acceleration over time. However, Figure 1(c) reveals considerable dispersion around a likely quasilinear increase in the Ashanti Region.

In summary, the only consistent aspect is the overall increase in the frequency of cesarean births (*N*_*i*_) over time (*t*). Regions exhibiting convexity at later times may imply a potential exponential increase, raising concerns about severity if the trend persists. The unnatural pattern also suggests a potential shift and slowdown in the future (that is, for *t* > 120 months).

A universal model is imperative considering the unique characteristics of each region. Deviations from the common relationship on specific dates require interpretation to elucidate the dynamics of cesarean in individual regions. To mitigate dispersion and random fluctuations, we propose two sequential steps.

The Greater Accra and Ashanti regions are known to have the highest numbers of cesarean births, which might be attributed to higher population density, better healthcare facilities, or other factors. The Northern region has a relatively lower number of cesarean births compared to the more populous regions in the South. Variations in cesarean birth numbers could be influenced by factors such as healthcare infrastructure, socioeconomic conditions, and regional population sizes. These findings can inform healthcare planning, resource allocation, and policy decisions to ensure adequate maternal care and childbirth services in different regions of Ghana. The Brong-Ahafo region, depicted in Figure 1(a), exhibits a convex shape, indicating a possible deceleration in cesarean births beyond 2017. There is a likelihood of an exponential increase in later periods, raising concerns about the seriousness of the trend if left unchecked. This underscores the need for vigilant monitoring and potential interventions to effectively address the observed pattern. In the Upper West Region, an upward trend with a concave appearance implies a potential acceleration in cesarean births over time. Understanding the contributing factors is crucial to effective healthcare planning. The Ashanti region (Figure 1(c)) shows significant dispersion, which presents a challenge in identifying a clear pattern. This complexity underscores the need for a nuanced approach and further investigation to accurately interpret the dynamics of cesareans in the region. The analysis reveals a consistent global increase in cesarean births over time, with regions that exhibit convexity suggesting a potential exponential increase, which demands attention and intervention. Concerns are raised about the unnatural nature of observed patterns and the possibility of a change and slowdown in the future (for *t* > 120 months). Emphasizing the importance of a universal model while considering regional characteristics, the analysis calls for interpreting specific deviation dates to fully comprehend cesarean dynamics. The two proposed steps are aimed at minimizing dispersion and fluctuations, striving for a more accurate representation of the trends of cesarean birth. In summary, the findings underscore the need for a region-specific approach in addressing various cesarean birth patterns. The insights are valuable for healthcare planning, resource allocation, and policy decisions, especially in regions with a high number of cesarean births such as Greater Accra and Ashanti. Continued monitoring and intervention are considered crucial to ensure optimal maternal care in different regions of Ghana.

### 3.2. Frequency of Cesarian Births in Ghana

**Figure 2:**
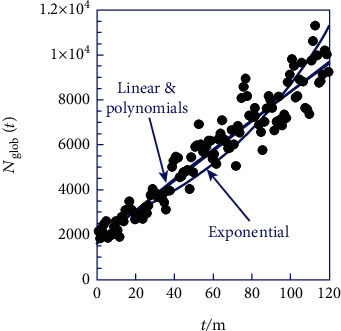
Frequency of cesarian births *N*_glob_(*t*) recorded monthly from 2008 to 2017, covering the entire country of Ghana (Supporting Information).

In the initial phase, we have visually depicted the occurrence of cesarean births, denoted as *N*_glob_(*t*), across the 10 regions for each month (*t*) over the entire decade, spanning from 2008 to 2017, as expressed in Equation (4). The graphical representation of this frequency is presented in Figure 2.

(4) $$\begin{array}{c} N_{\text{glob}}\left(t\right)=\overset{i=10}{\underset{i=1}{\sum }}N_{i}\left(t\right) \end{array}$$

Upon examination of Figure 2, a noticeable reduction in the previously observed dispersions in Figure 1 is evident. This prompts the consideration of a potential unified model, where each region exhibits specific deviations from the common relationship, a topic worthy of discussion. The concave pattern of scatter points in Figure 2 motivates us to explore three distinct mathematical forms, as outlined in Table 2.

Table 2 demonstrates that the exponential form not only presents a lower correlation coefficient (*R*) but also exhibits a more noticeable curvature compared to the general trend of scatter points depicted in Figure 2. As a result, we will discard this suggested mathematical form, primarily due to its rapid divergence, which is considered unnatural. The *R*-square shows a marginal improvement between the linear model and the second-degree polynomial, indicating a slight deviation toward quasilinearity. Furthermore, the last polynomial coefficient in Table 2 has a negatively low value (−0.049925), suggesting a potential slowdown or the continuation of quasilinear behavior in the future (i.e., post-2017). Additionally, the *R*-squares of the second and third polynomials practically share identical values, rendering the concept of a third-order polynomial irrelevant. Therefore, it becomes imperative to explore quasilinearity disrupted by a mathematical function of minimal impact.

The exponential model suggests that the frequency of cesarean births follows an exponential growth pattern over time. The correlation coefficient of 0.868076 indicates a reasonably good fit, suggesting that the exponential model captures a significant portion of the observed variation. The linear model proposes a straight-line relationship between the frequency of cesarean births and time. With an *R*-squared value of 0.910513, this model demonstrates a strong linear correlation, which implies that a linear trend can explain a substantial proportion of observed variability. The second-degree polynomial model introduces a quadratic term, allowing for a more flexible curve. The correlation coefficient of 0.911012 indicates an improvement in fit compared to the linear model, suggesting that the curvature captured by the quadratic term contributes to explaining the observed data. The third-degree polynomial model further extends the flexibility by including a cubic term. The correlation coefficient of 0.911021 shows a marginal improvement over the second-degree polynomial, indicating that the cubic term has a limited impact in explaining the variability. In summary, all proposed models (exponential, linear, second-degree polynomial, and third-degree polynomial) exhibit relatively high *R-*square values, suggesting that each model captures a substantial portion of the observed variation in the frequency of cesarean births over the specified time period.

### 3.3. Cumulative Frequency of Cesarian Births in Ghana

**Figure 3:**
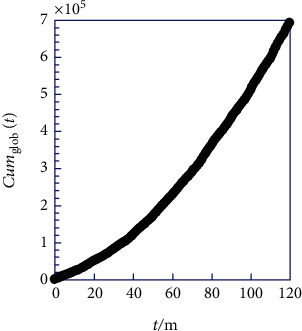
Cumulative frequency of cesarian births recorded Cum_glob_ (*t*) for each month from 2008 to 2017 for all counties in Ghana (Supporting Information).

As the next step, we have taken the initiative to graphically represent the cumulative frequency, Cum_glob_(*t*) (Equation (5)), in Figure 3. This cumulative frequency is considered for all 10 regions at each month throughout the entire decade from 2008 to 2017 (see the Supporting Information section (available here)). It is assumed that we have zero cumulative frequency at zero month to facilitate mathematical continuity. This allows us to compute the derivative of Cum_glob_(*t*) concerning time (Equation (6)) to determine (*N*_glob_) values over time (*t* > 0).

(5) $$\begin{array}{c} {\text{Cum}}_{\text{glob}}\left(t\right)=\overset{\theta =t}{\underset{\theta =1}{\sum }}N_{\text{glob}}\left(\theta \right) \end{array}$$

where (*θ*) represents the time in months.

In this context, we witness the elimination of dispersion and the undulating pattern, paving the way for the exploration of a model that closely mirrors physical reality or the phenomenon under investigation. Subsequently, we can formulate the empirical expression for the frequency of cesarean birth, *N*_glob_(*t*), over time (*t*) by differentiating (Equation (5)) with respect to time (*t*). It is assumed that the cumulative frequency, Cum_glob_(*t*), intersects zero-Cum at zero months to ensure precise values at the temporal origin (Equation (6)). Notably, this technique bears resemblance to the Riemann integration, but in reverse.

(6) $$\begin{array}{c} N_{\text{glob}}\left(t\right)=\frac{d{\text{Cum}}_{\text{glob}}\left(t\right)}{\text{dt}} \end{array}$$

Based on the key inferences from the models in Table 2, suggesting that quasilinear behavior is likely for the frequency of cesarean births, *N*_glob_(*t*), and consequently for the cumulative frequency, Cum_glob_(*t*), as its primitive function, a second-degree polynomial is considered suitable. Applying the least squares method to the cumulative frequency data, Cum_glob_(*t*), yields optimal parameter values for the second-degree polynomial expressed in Equation (7).

(7) $$\begin{array}{c} \text{Cu}{\text{m}}_{\text{glob}}\left(t\right)=–1562.6+1833.74\cdot t+32.9723\cdot t^{2} \end{array}$$

This polynomial model has an *R*-square value of 0.999848. Subsequently, in accordance with Equation (6), the derivative of Equation (7) gives Equation (8).

(8) $$\begin{array}{c} N_{\text{glob}}\left(t\right)=1833.74+65.9446\cdot t \end{array}$$

Table 3 presents a comparison of the actual cumulative frequency values (Cum_glob_(*t*)) and the frequency of cesarean births (*N*_glob_(*t*)) with those computed using Equations (7) and (8), respectively. This comparison is made at the beginning and end of the period spanning from 2008 to 2017, covering the entire country of Ghana.

The visual representation in the Supporting Information section initially suggests overall satisfaction. However, the comparison in Table 3 reveals significant deviations in the calculated cumulative frequency (Cum_glob_(*t*)) values, particularly for low time values, such as the beginning of the 2008 period. In particular, at zero time, a non-null value is observed, and for the first month (*t* = 1), the calculated value of 304.11 differs markedly from the actual value of 2122, with a relative deviation of approximately 85.7%.

To address these disparities, as a third step, we investigated the relationship between cumulative frequency (Cum_glob_(*t*)) and time (*t*). Taking into account the mathematical property associated with zero time, we depicted the ratio (Cum_glob_(*t*)/*t*) in the Supporting Information section to observe the trend of the scatter point and extrapolate the intercept to the ordinate, which corresponds to that of *N*_glob_ at zero time (*N*_0,glob_) for December 2007. This intercept should be slightly less than *N*_1,glob_ = 2122 in the first month (*t* = 1), based on the trend observed in Figure 2.

On careful examination in the Supporting Information section, a subtle positive deviation from quasilinearity is evident, indicating the potential presence of an oblique asymptote at high time values. The scatter points suggest that the most fitting representation for this deviation might be an exponential form with an oblique asymptote (quasilinearity).

It is crucial to note, however, that dividing by time (*t*) in the cumulative frequency (Cum_glob_(*t*)/*t*) accentuates fluctuations at low time values (Supporting Information) and decreases them at high time values (Supporting Information). Consequently, we also considered exploring the complementary cumulative frequency Cum′glob′ (Equation (9)), graphically representing the second area (Supporting Information) within the time range (*t*, *t*_*f*_ = 120).

(9) $$\begin{array}{c} \text{Cu}{\text{m}}_{\text{glob}}^{\prime }\left(t\right)=\overset{\theta =t_{f}}{\underset{\theta =t+1}{\sum }}N_{\text{glob}}\left(\theta \right) \end{array}$$

where *t*_*f*_ represents the final time (*t*_*f*_ = 120 months). So, given Equations (1), (2), (4), and (5), we can write the following relation:

(10) $$\begin{array}{c} {\text{Cum}}_{\text{glob}}\left(t\right)+\text{Cu}{\text{m}}_{\text{glob}}^{\prime }\left(t\right)=N_{\text{tot}}=692045 \end{array}$$

The graphical representation of the complementary cumulative frequency Cum_glob_′(*t*) is shown in the Supporting Information section, presented both as a function of (*t*) and as a function of (*t*_*f*_–*t*). Similar to the Supporting Information section, where Cum_glob_(*t*) exhibits near linearity, except for low time values (t) where a noticeable positive deviation from linearity is observed, we have plotted (Cum′_glob_(*t*)/(*t*_*f*_–*t*) as a function of (*t*_*f*_–*t*) in the Supporting Information section, aiming to detect potential pronounced fluctuations at low time values (*t*_*f*_–*t*). Indeed, we observe quasilinearity preceded by a certain perturbation at the onset of (*t*_*f*_–*t*), occurring on both sides of the linearity extension. This implies that the disturbances noted for low values of time (*t*) in the Supporting Information section are likely attributed not only to the mathematical property of division by (*t*), but potentially also to a phenomenon or a specific cause that rapidly diminished and disappeared over time.

Table 3 presents the variation in both the cumulative frequency (Cum_glob_(*t*)) and the frequency of cesarean births (*N*_glob_(*t*)) over time (*t*) for each month, recorded at the beginning and end of the period 2008 to 2017 for the entire country of Ghana. The table includes the actual values, the values calculated using specific equations (Equation (7) and Equation (8)), and the percentage errors between the calculated and actual values.

Cumulative frequency errors range from −85.669% to 0.24986%, with an arithmetic mean error of −1.998%. These errors indicate the percentage difference between the calculated and actual cumulative frequencies at different time points. Overall, the average error suggests a slight underestimation of the cumulative frequencies calculated. The frequency of cesarean birth errors varies from −15.819% to 22.092%, with an arithmetic mean error of 2.9147%. These errors represent the percentage difference between the calculated and actual frequencies of cesarean births. Positive and negative errors suggest both underestimation and overestimation at different time points. The average error indicates a slight overestimation in the calculated frequencies of cesarean births. The table also includes values at time point 0, marked as a mathematical assumption, and shows discrepancies between the calculated and actual values.

In summary, the table provides a comprehensive overview of the precision of calculated cumulative frequencies and frequencies of cesarean births over the specified time period. The percentage errors highlight the deviations between the calculated and actual values, and the arithmetic mean errors give a sense of the overall accuracy of the calculations. The findings suggest a general trend of underestimation in cumulative frequencies and a slight overestimation in the frequencies of cesarean births in the context of the provided equations.

### 3.4. Quadratic Model Endowed With Exponential

Before starting with the suggested exponential model, researchers starting out in the field of empirical modeling used GeoGebra Classique software and presented in the Supporting Information section, other possible mathematical forms (Supporting Information), which were abandoned for certain discussed reasons (Supporting Information).

Taking into account the previous discussions, we can suggest the following empirical expressions.

(11) $$\begin{array}{c} \frac{{\text{Cum}}_{\text{glob}}\left(t\right)}{t}=\frac{1}{2}A_{0}t+B_{0}+Be^{-t/\tau } \end{array}$$

Then,

(12) $$\begin{array}{c} {\text{Cum}}_{\text{glob}}\left(t\right)=\frac{1}{2}A_{0}t^{2}+B_{0}t+Bte^{-t/\tau } \end{array}$$

Given the derivation property in Equation (6), we can then write the following:

(13) $$\begin{array}{c} N_{\text{glob}}\left(t\right)=A_{0}t+B_{0}+B\left(1-\frac{t}{\tau }\right)e^{-t/\tau } \end{array}$$

**Figure 4:**
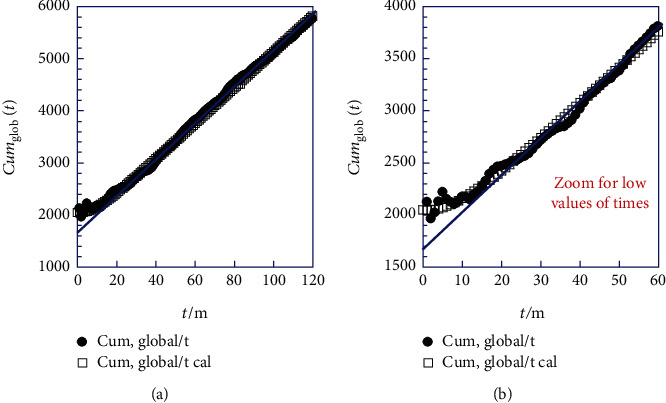
Comparison between the actual and calculated ratio (Cum_glob_/*t*) over the time (*t*) recorded for each month (a) from 2008 to 2017 and (b) Zoom for the first 60-month period, for all over the country of Ghana. Calculated with Equation (11) and solid line: oblique asymptote (*N*_asymp_(*t*) = 1697.85 + 34.2952 · *t*) (Equation (15)).

It is evident that the plot of the ratio of cumulative frequency to time (Cum_glob_(*t*)/*t*) in Figure 4 accurately depicts this quasilinearity deviation.

As a first and swift analysis of Figure 4, we can give, as an approximative interpretation, the following comment: We can subdivide the phenomenon of increased frequency of cesarian births *N*_glob_(*t*) into two regimes, such as transient *N*_trans_(*t*) and permanent *N*_perm_(*t*), where after a few months the steady state quickly establishes itself as a quasilinear phenomenon *N*_glob_ (*t*) *N*_perm_ (*t*). Equations (14), (15), and (16) express clearly the subdivision of Equation (13).

(14) $$\begin{array}{c} N_{\text{glob}}\left(t\right)=N_{\text{trans}}\left(t\right)+N_{\text{perm}}\left(t\right) \end{array}$$

(15) $$\begin{array}{c} N_{\text{asymp}}\left(t\right)=N_{\text{perm}}\left(t\right)=A_{0}t+B_{0} \end{array}$$

(16) $$\begin{array}{c} N_{\text{trans}}\left(t\right)=B\left(1-\frac{t}{\tau }\right)e^{-t/\tau } \end{array}$$

The transient phase (Equation (16)) is based on a short period with approximately negligible deviation from quasilinearity. We encountered a small convergence problem when applying the Python code (Supporting Information) to the least-squares method. For the different initialization levels of the four free adjustable parameters of the proposed model (Equation (13)), the values of (*τ*) become more or less random, while the value of (*B*) tends toward zero when we increase the number of iterations, thus forcing the phenomenon of being described only by the permanent regime (quasilinearity, Equation (15)).

To fix this constraint of empirical modeling, we have proceeded with some manual steps as follows:

i. We have omitted the data of the 24 first times in the Supporting Information section to avoid the deviation from linearity (Figure 4) and fitted the rest in linear regression to bring to light the permanent and dominant regime (quasilinearity, Equation (15)). We then obtain the following result:
  (17) $$\begin{array}{c} \frac{{\text{Cum}}_{\text{glob}}\left(t\right)}{t}\approx \frac{1}{2}A_{0}t+B_{0}=34.2952∙t+1697.85 \end{array}$$

With a correlation coefficient equal to *R* = 0.998902 and for *t* < 25. We can then consider with good approximation that the values obtained of (*A*_0_ and *B*_0_) are the optimal values of the oblique asymptote (Table 4).

ii. We have combined Equations (11) and (17) and fitted all the data in the Supporting Information section in exponential form, leaving (*A*_0_ and *B*_0_) as fixed parameters and (*B* and *τ*) as free adjustable parameters. We then obtain the following result:
  (18) $$\begin{array}{c} \frac{{\text{Cum}}_{\text{glob}}\left(t\right)}{t}-\left(\frac{1}{2}A_{0}t+B_{0}\right)=Be^{-t/\tau }=400.192∙e^{-0.120117∙t} \end{array}$$

With a correlation coefficient equal to *R* = 0.868064 and for *t* 1. We can then consider with good approximation that the values obtained of (*B* and *τ*) are the optimal values of the exponential part or the deviation from the quasilinearity (Table 4).

iii. Finally, regarding the previously discussed constraint of iteration divergence, due to the feeble deviation from linearity for a few months, we have forced the fit to take more importance of this nondominant effect of the transient phase. To do this, we vary the multiplicity (*w*) of the first part of the data, including the deviation (1 < *t* < 12), by using the parameters values obtained (*A*0 = 68.5904, *B*0 = 1697.85, *B* = 400.192 and *τ* = 8.325) as initialization to find new globally optimal values, thus leaving the four parameters (*A*_0_, *B*_0_, *B*, and *τ*) as free and adjustable in the operation of the least squares method using Python code (Supporting Information). The Supporting Information section gives the optimal values obtained for (*A*_0_, *B*_0_, *B*, and *τ*) for different (*w*) repetitions as a statistical weight, ranging from once to 20 times. The Supporting Information section shows the variation of parameters for different weights (*w*). Given that the exponential parameter (*B*) is in some way an amplitude of the transient effect, which is a short and nondominant phenomenon and which passes through a minimum in the Supporting Information section at *w* = 4, we saw fit to adopt as definitive optimal parameters for the present study those of repetition (*w* = 4). Table 4 summarizes the main results from the detailed Supporting Information.

The relative standard deviation (*σ*_rel_, defined in the Supporting Information section) is provided for the initial parameters and variations under different duplication weights. This measure quantifies the percentage variation relative to the mean, offering insight into the stability of the estimated parameters.

In summary, the table outlines the optimal parameter values and their variations based on different statistical weights (duplication levels). These values are crucial for understanding the behavior of the model and its ability to capture the underlying trends in the data. The inclusion of the relative standard deviation provides additional information on the stability and precision of the estimated parameters.

In addition, the exponential exponent (*τ*), which in some way reflects the rapidity of establishing the quasilinear permanent regime, has a value (8.546) very close to the pregnancy period, to within 5%.

Then, numerical applications of our proposed model (Equation (13)) require the use of the following convenient simple formulas for any estimating results into the studied time range (interpolation) or for predicting values before or after the studied time range if the phenomenon keeps the same behavior (extrapolation):

(19) $$\begin{array}{c} N_{\text{glob}}\left(t\right)=68.5533∙t+1698.834+392.870\left(1-\frac{t}{8.546}\right)e^{-t/8.546} \end{array}$$

Finally, we warn that this quasilinearity model (Equation (15)) cannot allow future values to be predicted (extrapolated) for long. This is because the phenomenon of augmentation cannot continue at the same rate until infinity. It must slow down to reach a certain stability or pseudoplateau. To do this, we recommend modifying the linear model for future data (i.e., after 2017) by incorporating an additional mathematical expression that reflects this very probable slowdown.

## 4. Findings

The analysis of cesarean birth patterns across 10 regions of Ghana from 2008 to 2017 elucidates nuanced trends with significant implications for maternal healthcare. The total number of cesarean births (Ntot) recorded in Ghana over the 10-year period was calculated to be 692,045, with a relative proportion of 2.545% relative to the median population and approximately 5.1% relative to the female population. Notably, the Central Region exhibits a relatively stable trend, suggesting consistent access to maternal healthcare services. Conversely, the Eastern Region demonstrates a gradual increase in cesarean births, hinting at improving healthcare infrastructure and utilization. High cesarean birth rates in the Greater Accra Region mirror urbanization and advanced healthcare facilities, while the Northern Region's lower rates may stem from socioeconomic factors and cultural practices. The Upper East Region faces challenges in healthcare access, evident in its lower cesarean birth rates, whereas the Volta Region presents moderate rates, indicating balanced healthcare access. The Western Region's increasing trend suggests improving healthcare infrastructure. Graphical representations unveil diverse scatter point patterns across regions, with convex and concave trends observed, warranting vigilant monitoring and interventions. Mathematical modeling, including polynomial and quadratic models, captures substantial variation in cesarean birth frequencies, providing insights for predicting future trends and informing policy decisions to ensure optimal maternal healthcare outcomes nationwide.

Various mathematical models were proposed to capture the trends in cesarean births over time, including exponential, linear, and polynomial models. The second-degree polynomial model and the third-degree polynomial model demonstrated relatively high correlation coefficients, suggesting that they capture a significant portion of the observed variation in cesarean birth frequencies. The cumulative frequency of cesarean births was also analyzed, and a second-degree polynomial model was found to closely approximate the observed data.

A quadratic model endowed with an exponential term was proposed to account for deviations from linearity and capture the transient and permanent phases of cesarean birth frequency trends. The optimal parameters of the proposed model were determined through a series of iterations, considering different statistical weights. The relative standard deviation was used to assess the stability and precision of the estimated parameters. The final model provided a convenient model for estimating cesarean birth frequencies over time within the studied range and for predicting values outside the range, with consideration for the probable slowdown in the increase of cesarean births in the future.

## 5. Conclusions

In summary, the research employs a systematic and comprehensive empirical methodology to analyze cesarean birth trends in Ghana, considering regional variations and proposing a refined model that incorporates both quadratic and exponential components. A comprehensive analysis of C-section deliveries in Ghana spanning 2008 to 2017 reveals distinct regional patterns and a global increase in C-section births. The study underscores the importance of understanding the reasons behind scheduled and emergency C-sections, focusing on responses to various maternal and fetal conditions. Regional variations in C-section numbers are observed, particularly in the Greater Accra and Ashanti regions, possibly influenced by factors such as population density, healthcare facilities, and socioeconomic conditions. The study advocates region-specific healthcare planning to ensure optimal maternal care. Empirical models, including linear, quadratic, and exponential components, offer insights into the dynamics of the C-section over time. The quadratic model, incorporating an exponential deviation, captures quasilinear behavior with transient and permanent phases. An oblique asymptote enhances the model's accuracy in representing short-term fluctuations in C-section frequency, raising concerns about unnatural patterns and the potential for future changes. Continuous monitoring and interventions are considered essential. The region-specific approach is emphasized for effective healthcare planning, considering unique regional characteristics. Limitations include simplification of real-world complexities through mathematical modeling and potential data inaccuracies. Despite limitations, the study contributes valuable information on Ghana's C-section trends, calling for ongoing monitoring, intervention, and region-specific strategies for optimal maternal care. Future research could explore additional influencing factors and refine models to enhance accuracy and applicability.

## 6. Limitations and Recommendations

• The research acknowledges limitations, including data simplification through mathematical modeling and potential data inaccuracies.
• The findings underscored the importance of region-specific approaches in addressing cesarean birth patterns in Ghana.
• The need for caution in extrapolating future values is highlighted, suggesting modifications to the linear model to reflect the expected slowdown in the increase in cesarean births.

## Figures and Tables

**Table 1:** List of the 10 regions (Supporting Information) in Ghana and the corresponding total cesarian births (*N*_*i*,tot_).

**i**	**1**	**2**	**3**	**4**	**5**	**6**	**7**	**8**	**9**	**10**
Region	Brong-Ahafo	Central	Eastern	Greater Accra	Northern	Upper East	Upper West	Volta	Western	Ashanti
Abbreviation	BA	CNT	EAST	ACC	NTH	UE	UW	VLT	WST	ASH
*N*_*i*,tot_	74002	66676	76105	184307	37231	17739	12687	49938	65090	108270

**Table 2:** (*R*) square related to some empirical expressions suggested for the variation of the frequency of cesarian births *N*_glob_(*t*) over the time (*t*) recorded for each month from 2008 to 2017 for all of Ghana (Supporting Information).

**Model form**	**Expression**	**Correlation coefficient (** **R** ^2^ **)**
Exponential	*N*_glob_(*t*) = 2374.68 · e^0.0129756·*t*^	0.868076
Linear	*N*_glob_(*t*) = 1769.24 + 66.0794 · *t*	0.910513
Second-degree polynomial	*N*_glob_(*t*) = 1646.40 + 72.1203 · *t*–0.049925 · *t*^2^	0.911012
Third-degree polynomial	*N*_glob_(*t*) = 1626.71 + 74.1312 · *t*–0.0913001 · *t*^2^ + 0.000227962 · *t*^3^	0.911021

**Table 3:** Variation of the cumulative frequency Cum_glob_(*t*) and the frequency of cesarian births *N*_glob_(*t*) over the time (*t*) recorded for each month (at the beginning and end of the period) from 2008 to 2017 for all counties in Ghana (Supporting Information).

**Time (*t*)**	**C** **u** **m**_**g** **l** **o** **b**_(**t**)		**Error (%)**	**N**_**g** **l** **o** **b**_(**t**)		**Error (%)**
**(month)**	**Actual**	**Calculated (Equation** (7)**)**	**100 (calc-exp)/exp**	**Actual**	**Calculated (Equation** (8)**)**	**100 (calc-exp)/exp**
0	0^a^	−1562.6	—	—	1833.7	—
1	2122	304.11	−85.669	2122	1899.7	−10.477
2	5419	2236.8	−42.969	3297	1965.6	9.2016
3	10265	4235.4	−30.190	4846	2031.6	−5.2879
4	16863	6299.9	−25.988	6598	2097.5	−14.212
5	25250	8430.4	−23.927	8387	2163.5	−15.819
6	34710	10627	−17.672	9460	2229.4	22.092
7	45415	12889	−13.245	10705	2295.4	17.771
...	...	...	...	...	...	...
115	644788	645376.2	0.091222	8727	9417.4	7.9107
116	653579	654826.5	0.19087	8791	9483.3	7.8753
117	662687	664342.8	0.24986	9108	9549.3	4.8447
118	672856	673925.0	0.15888	10169	9615.2	−5.4459
119	682839	683573.2	0.10752	9983	9681.1	−3.0237
120	692045	693287.3	0.17951	9206	9747.1	5.8776
	Arithmetic mean		−1.998%	Arithmetic mean		2.9147%

^a^Mathematical assumption.

**Table 4:** Variation of the optimal values of (*A*_0_, *B*_0_, *B*, and *τ*) with the main statistical weight (*w*).

**Duplication (** **w** **)**	**A**_0_	**B**_0_	**B**	**τ** ** (m)**	**σ**_**r** **e** **l**_ ** (%)** ^a^
Oblique asymptote (linear part, Equation (17))	68.5904	1697.85	—	—	—
Deviation to the linearity (exponential part, Equation (18))	—	—	400.192	8.325	—
Initial parameters	68.5904	1697.85	400.192	8.325	1.5333
Duplication, *w* = 1	68.4362	1704.14	397.798	7.965	1.5282
Duplication, *w* = 4	**68.5533**	**1698.834**	**392.870**	**8.546**	1.5182

^a^Relative standard deviation, *σ*_rel_, defined in the Supporting Information section.

## Data Availability

The data used to support the findings of this study are available from the corresponding author upon request.

## Supporting Information

Supporting Information Additional supporting information can be found online in the Supporting Information section. Supporting information on the methodological and additional results of the examining cesarean section rates in Ghana's 10 regions over a decade of a comprehensive national investigation.
