# Supplementary material for: Examining Cesarean Section Rates in Ghana's 10 Regions Over a Decade a Comprehensive National Investigation
Source: Biomed Res Int. 2024 Nov 9;2024:3774435. doi: 10.1155/2024/3774435 (PMC11568890; doi:10.1155/2024/3774435)
Supplement: Supporting Information — Additional supporting information can be found online in the Supporting Information section. Supporting information on the methodological and additional results of the examining cesarean section rates in Ghana's 10 regions over a decade of a comprehensive national investigation. [file 3774435.f1.docx]

**Supplementary Materials**

**Part A. Case of the overall the Country.**


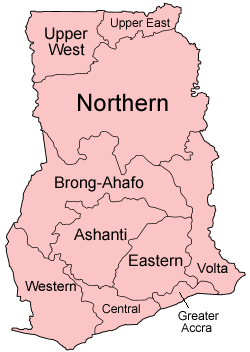


**Scheme 1S**. Ghana is divided into 10 Administrative Regions sub-divided into a total of 216 Districts. From link: <https://mymaps3.blogspot.com/2021/09/ghana-map-regions.html>

**Table 1S**. Data for frequency of caesarian births *N_glob_*(*t*) and derived properties recorded for each month from 2008 to 2017 for all over the Country of Ghana.

| Month  (*t*) | Year | Month # | Date | *N_glob_*(*t*) | *Cum_glob_*(*t*) | *Cum’_glob_*(*t*) | *Cum_glob_* / *t* |
| --- | --- | --- | --- | --- | --- | --- | --- |
| 0 | 200700 | 12 | - | - | 0 | 692045 | - |
| 1 | 200800 | 1 | 08-janv | 2122 | 2122 | 689923 | 2122 |
| 2 | 200800 | 2 | 08-févr | 1800 | 3922 | 688123 | 1961 |
| 3 | 200800 | 3 | 08-mars | 2145 | 6067 | 685978 | 2022,333 |
| 4 | 200800 | 4 | 08-avr | 2445 | 8512 | 683533 | 2128 |
| 5 | 200800 | 5 | 08-mai | 2570 | 11082 | 680963 | 2216,4 |
| 6 | 200800 | 6 | 08-juin | 1826 | 12908 | 679137 | 2151,333 |
| 7 | 200800 | 7 | 08-juil | 1949 | 14857 | 677188 | 2122,429 |
| 8 | 200800 | 8 | 08-août | 1957 | 16814 | 675231 | 2101,75 |
| 9 | 200800 | 9 | 08-sept | 2327 | 19141 | 672904 | 2126,778 |
| 10 | 200800 | 10 | 08-oct | 2572 | 21713 | 670332 | 2171,3 |
| 11 | 200800 | 11 | 08-nov | 2170 | 23883 | 668162 | 2171,182 |
| 12 | 200800 | 12 | 08-déc | 1865 | 25748 | 666297 | 2145,667 |
| 13 | 200900 | 1 | 09-janv | 2770 | 28518 | 663527 | 2193,692 |
| 14 | 200900 | 2 | 09-févr | 2542 | 31060 | 660985 | 2218,571 |
| 15 | 200900 | 3 | 09-mars | 3049 | 34109 | 657936 | 2273,933 |
| 16 | 200900 | 4 | 09-avr | 3090 | 37199 | 654846 | 2324,938 |
| 17 | 200900 | 5 | 09-mai | 3448 | 40647 | 651398 | 2391 |
| 18 | 200900 | 6 | 09-juin | 3081 | 43728 | 648317 | 2429,333 |
| 19 | 200900 | 7 | 09-juil | 2981 | 46709 | 645336 | 2458,368 |
| 20 | 200900 | 8 | 09-août | 2637 | 49346 | 642699 | 2467,3 |
| 21 | 200900 | 9 | 09-sept | 2737 | 52083 | 639962 | 2480,143 |
| 22 | 200900 | 10 | 09-oct | 2864 | 54947 | 637098 | 2497,591 |
| 23 | 200900 | 11 | 09-nov | 2928 | 57875 | 634170 | 2516,304 |
| 24 | 200900 | 12 | 09-déc | 2657 | 60532 | 631513 | 2522,167 |
| 25 | 201000 | 1 | 10-janv | 3168 | 63700 | 628345 | 2548 |
| 26 | 201000 | 2 | 10-févr | 2930 | 66630 | 625415 | 2562,692 |
| 27 | 201000 | 3 | 10-mars | 3263 | 69893 | 622152 | 2588,63 |
| 28 | 201000 | 4 | 10-avr | 3711 | 73604 | 618441 | 2628,714 |
| 29 | 201000 | 5 | 10-mai | 4003 | 77607 | 614438 | 2676,103 |
| 30 | 201000 | 6 | 10-juin | 3737 | 81344 | 610701 | 2711,467 |
| 31 | 201000 | 7 | 10-juil | 3779 | 85123 | 606922 | 2745,903 |
| 32 | 201000 | 8 | 10-août | 3631 | 88754 | 603291 | 2773,563 |
| 33 | 201000 | 9 | 10-sept | 3711 | 92465 | 599580 | 2801,97 |
| 34 | 201000 | 10 | 10-oct | 3566 | 96031 | 596014 | 2824,441 |
| 35 | 201000 | 11 | 10-nov | 3407 | 99438 | 592607 | 2841,086 |
| 36 | 201000 | 12 | 10-déc | 3072 | 102510 | 589535 | 2847,5 |
| 37 | 201100 | 1 | 11-janv | 3937 | 106447 | 585598 | 2876,946 |
| 38 | 201100 | 2 | 11-févr | 3919 | 110366 | 581679 | 2904,368 |
| 39 | 201100 | 3 | 11-mars | 4974 | 115340 | 576705 | 2957,436 |
| 40 | 201100 | 4 | 11-avr | 5244 | 120584 | 571461 | 3014,6 |
| 41 | 201100 | 5 | 11-mai | 5431 | 126015 | 566030 | 3073,537 |
| 42 | 201100 | 6 | 11-juin | 5389 | 131404 | 560641 | 3128,667 |
| 43 | 201100 | 7 | 11-juil | 4517 | 135921 | 556124 | 3160,953 |
| 44 | 201100 | 8 | 11-août | 4559 | 140480 | 551565 | 3192,727 |
| 45 | 201100 | 9 | 11-sept | 4746 | 145226 | 546819 | 3227,244 |
| 46 | 201100 | 10 | 11-oct | 4770 | 149996 | 542049 | 3260,783 |
| 47 | 201100 | 11 | 11-nov | 4832 | 154828 | 537217 | 3294,213 |
| 48 | 201100 | 12 | 11-déc | 4004 | 158832 | 533213 | 3309 |
| 49 | 201200 | 1 | 12-janv | 5411 | 164243 | 527802 | 3351,898 |
| 50 | 201200 | 2 | 12-févr | 4749 | 168992 | 523053 | 3379,84 |
| 51 | 201200 | 3 | 12-mars | 5866 | 174858 | 517187 | 3428,588 |
| 52 | 201200 | 4 | 12-avr | 5931 | 180789 | 511256 | 3476,712 |
| 53 | 201200 | 5 | 12-mai | 6878 | 187667 | 504378 | 3540,887 |
| 54 | 201200 | 6 | 12-juin | 5993 | 193660 | 498385 | 3586,296 |
| 55 | 201200 | 7 | 12-juil | 5660 | 199320 | 492725 | 3624 |
| 56 | 201200 | 8 | 12-août | 5602 | 204922 | 487123 | 3659,321 |
| 57 | 201200 | 9 | 12-sept | 5894 | 210816 | 481229 | 3698,526 |
| 58 | 201200 | 10 | 12-oct | 6146 | 216962 | 475083 | 3740,724 |
| 59 | 201200 | 11 | 12-nov | 6149 | 223111 | 468934 | 3781,542 |
| 60 | 201200 | 12 | 12-déc | 5350 | 228461 | 463584 | 3807,683 |
| 61 | 201300 | 1 | 13-janv | 5547 | 234008 | 458037 | 3836,197 |
| 62 | 201300 | 2 | 13-févr | 5112 | 239120 | 452925 | 3856,774 |
| 63 | 201300 | 3 | 13-mars | 6215 | 245335 | 446710 | 3894,206 |
| 64 | 201300 | 4 | 13-avr | 6466 | 251801 | 440244 | 3934,391 |
| 65 | 201300 | 5 | 13-mai | 7060 | 258861 | 433184 | 3982,477 |
| 66 | 201300 | 6 | 13-juin | 6216 | 265077 | 426968 | 4016,318 |
| 67 | 201300 | 7 | 13-juil | 6076 | 271153 | 420892 | 4047,06 |
| 68 | 201300 | 8 | 13-août | 5803 | 276956 | 415089 | 4072,882 |
| 69 | 201300 | 9 | 13-sept | 5828 | 282784 | 409261 | 4098,319 |
| 70 | 201300 | 10 | 13-oct | 6630 | 289414 | 402631 | 4134,486 |
| 71 | 201300 | 11 | 13-nov | 5985 | 295399 | 396646 | 4160,549 |
| 72 | 201300 | 12 | 13-déc | 5021 | 300420 | 391625 | 4172,5 |
| 73 | 201400 | 1 | 14-janv | 6804 | 307224 | 384821 | 4208,548 |
| 74 | 201400 | 2 | 14-févr | 6502 | 313726 | 378319 | 4239,541 |
| 75 | 201400 | 3 | 14-mars | 8056 | 321782 | 370263 | 4290,427 |
| 76 | 201400 | 4 | 14-avr | 8539 | 330321 | 361724 | 4346,329 |
| 77 | 201400 | 5 | 14-mai | 8926 | 339247 | 352798 | 4405,805 |
| 78 | 201400 | 6 | 14-juin | 8137 | 347384 | 344661 | 4453,641 |
| 79 | 201400 | 7 | 14-juil | 7273 | 354657 | 337388 | 4489,329 |
| 80 | 201400 | 8 | 14-août | 7233 | 361890 | 330155 | 4523,625 |
| 81 | 201400 | 9 | 14-sept | 7200 | 369090 | 322955 | 4556,667 |
| 82 | 201400 | 10 | 14-oct | 7621 | 376711 | 315334 | 4594,037 |
| 83 | 201400 | 11 | 14-nov | 7234 | 383945 | 308100 | 4625,843 |
| 84 | 201400 | 12 | 14-déc | 6855 | 390800 | 301245 | 4652,381 |
| 85 | 201500 | 1 | 15-janv | 6535 | 397335 | 294710 | 4674,529 |
| 86 | 201500 | 2 | 15-févr | 5732 | 403067 | 288978 | 4686,826 |
| 87 | 201500 | 3 | 15-mars | 6994 | 410061 | 281984 | 4713,345 |
| 88 | 201500 | 4 | 15-avr | 7592 | 417653 | 274392 | 4746,057 |
| 89 | 201500 | 5 | 15-mai | 8132 | 425785 | 266260 | 4784,101 |
| 90 | 201500 | 6 | 15-juin | 7954 | 433739 | 258306 | 4819,322 |
| 91 | 201500 | 7 | 15-juil | 7611 | 441350 | 250695 | 4850 |
| 92 | 201500 | 8 | 15-août | 6605 | 447955 | 244090 | 4869,076 |
| 93 | 201500 | 9 | 15-sept | 7002 | 454957 | 237088 | 4892,011 |
| 94 | 201500 | 10 | 15-oct | 8125 | 463082 | 228963 | 4926,404 |
| 95 | 201500 | 11 | 15-nov | 7081 | 470163 | 221882 | 4949,084 |
| 96 | 201500 | 12 | 15-déc | 6824 | 476987 | 215058 | 4968,615 |
| 97 | 201600 | 1 | 16-janv | 7311 | 484298 | 207747 | 4992,763 |
| 98 | 201600 | 2 | 16-févr | 7159 | 491457 | 200588 | 5014,867 |
| 99 | 201600 | 3 | 16-mars | 8748 | 500205 | 191840 | 5052,576 |
| 100 | 201600 | 4 | 16-avr | 9108 | 509313 | 182732 | 5093,13 |
| 101 | 201600 | 5 | 16-mai | 9784 | 519097 | 172948 | 5139,574 |
| 102 | 201600 | 6 | 16-juin | 9478 | 528575 | 163470 | 5182,108 |
| 103 | 201600 | 7 | 16-juil | 8078 | 536653 | 155392 | 5210,223 |
| 104 | 201600 | 8 | 16-août | 8134 | 544787 | 147258 | 5238,337 |
| 105 | 201600 | 9 | 16-sept | 8861 | 553648 | 138397 | 5272,838 |
| 106 | 201600 | 10 | 16-oct | 9618 | 563266 | 128779 | 5313,83 |
| 107 | 201600 | 11 | 16-nov | 8775 | 572041 | 120004 | 5346,178 |
| 108 | 201600 | 12 | 16-déc | 7620 | 579661 | 112384 | 5367,231 |
| 109 | 201700 | 1 | 17-janv | 7554 | 587215 | 104830 | 5387,294 |
| 110 | 201700 | 2 | 17-févr | 7345 | 594560 | 97485 | 5405,091 |
| 111 | 201700 | 3 | 17-mars | 9705 | 604265 | 87780 | 5443,829 |
| 112 | 201700 | 4 | 17-avr | 10577 | 614842 | 77203 | 5489,661 |
| 113 | 201700 | 5 | 17-mai | 11267 | 626109 | 65936 | 5540,788 |
| 114 | 201700 | 6 | 17-juin | 9952 | 636061 | 55984 | 5579,482 |
| 115 | 201700 | 7 | 17-juil | 8727 | 644788 | 47257 | 5606,852 |
| 116 | 201700 | 8 | 17-août | 8791 | 653579 | 38466 | 5634,302 |
| 117 | 201700 | 9 | 17-sept | 9108 | 662687 | 29358 | 5663,991 |
| 118 | 201700 | 10 | 17-oct | 10169 | 672856 | 19189 | 5702,169 |
| 119 | 201700 | 11 | 17-nov | 9983 | 682839 | 9206 | 5738,143 |
| 120 | 201700 | 12 | 17-déc | 9206 | 692045 | 0 | 5767,042 |

**Figure 1S.** Variation of the cumulative frequency *Cum_glob_*(*t*) and the frequency of caesarian births *N_glob_*(*t*) over the time (*t*) recorded for each month from 2008 to 2017 for all over the Country of Ghana. (●): actual; (□): calculated with (a): Eq. 7 and (b): Eq. 8, respectively. (Table 1S).

**Figure 2S.** Variation of the ratio (*Cum_glob_* / *t*) over the time (*t*) recorded for each month (a): from 2008 to 2017 and (b): Zoom for the first 50 months period, for all over the Country of Ghana (Table 1S).

**Figure 3S.** Frequency of caesarian births *N_glob_*(*θ*) recorded for each month from 2008 to 2017 for all over the Country of Ghana (Table 1S) and Riemann Areas corresponding to the cumulative frequency *Cum_glob_*(*t*) and the complementary cumulative frequency *Cum’_glob_*(*t*), respectively.

**Figure 4S.** Variation of the complementary cumulative frequency *Cum’_glob_*(*t*) recorded for each month from 2008 to 2017 for all over the Country of Ghana (Table 1S).

**Figure 5S.** Variation of the reduced complementary cumulative frequency *Cum’_glob_*(*t_f_-t*) / (*t_f_-t*) recorded for each month from 2008 to 2017 for all over the Country of Ghana (Table 1S)

Before starting with the suggested exponential model and, for young researchers starting out in the field of empirical modeling, we have used *GeoGebra Classique* Software for two other possible mathematical forms (Eqs. 1S and 2S), that were abandoned for certain disadvantages.

**First suggestion**: Hyperbolic form.

$\frac{{Cum}_{glob}\left( t \right)}{t}= A_{0}\cdot t+B_{0}+ \frac{\gamma}{\alpha\cdot t+\beta}$ (1S)


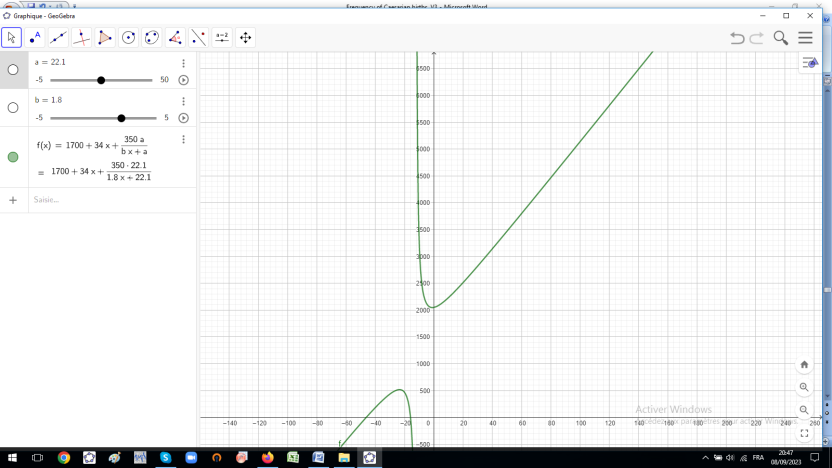


**Figure 6S.** Variation of the ratio (*Cum_glob_* / *t*) over the time (*t*) using *GeoGebra Classique* Software and (Eq. 1S).

By scanning the values of the adjustable parameters in real time, we observe the possible divergence due to the vertical asymptote very close to the time domain studied and an abnormal augmentation for high values of time.

**Second suggestion**: Logarithmic form.

$\frac{{Cum}_{glob}\left( t \right)}{t}= B_{0}-A_{0}(t+t_{0}) ln\frac{t_{0}}{t+t_{0}}$ (2S)


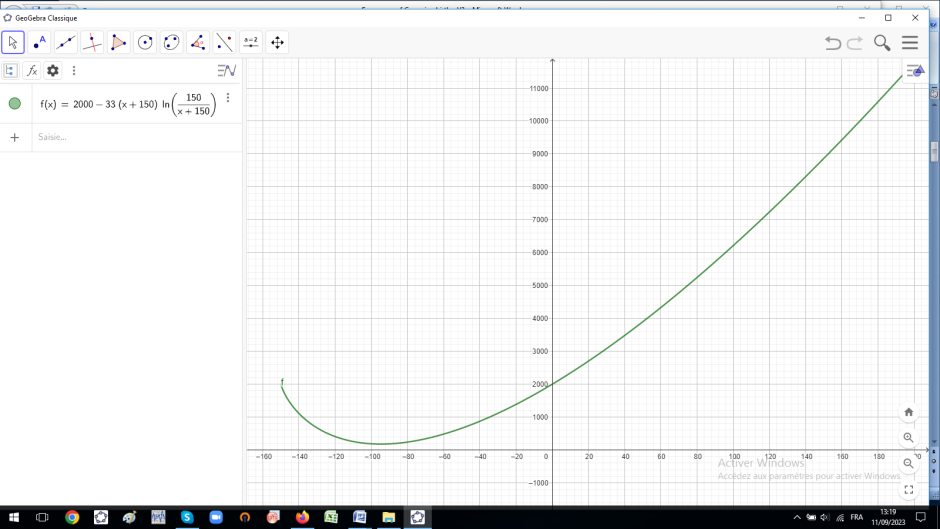


**Figure 7S.** Variation of the ratio (*Cum_glob_* / *t*) over the time (*t*) using *GeoGebra Classique* Software and (Eq. 2S).

By scanning the values of the adjustable parameters in real time, we observe the possible divergence due to the net absence of deviation to the linearity and an abnormal augmentation for high values of time due to the parabolic branch of this mathematical function.

**Scheme 2S**. Python code. Least square optimization adapted (personalized) for the suggested equation.

| with open("cumul_by_t.txt") as file:      DATA = tuple(map(float,file.read().split()))  from math import exp, sqrt  deff(A0,B0,B,Tau,t):      return1/2*A0*t + B0 + B*exp(-t/Tau)  defdelta(A0,B0,B,Tau,w):      returnsum((f(A0,B0,B,Tau,t)-val)**2fort,valinenumerate(DATA[:12],start=1))*w +\             sum((f(A0,B0,B,Tau,t)-val)**2fort,valinenumerate(DATA[12:],start=13))  defoptimize(A0,B0,B,Tau,w):      epsilon1 = A0/20      epsilon2 = B0/20      epsilon3 = B/20      epsilon4 = Tau/20      for_inrange(10000):          A0 = min((A0-epsilon1,A0,A0+epsilon1),key=lambdap:delta(p,B0,B,Tau,w))          B0 = min((B0-epsilon2,B0,B0+epsilon2),key=lambdap:delta(A0,p,B,Tau,w))          B = min((B-epsilon3,B,B+epsilon3),key=lambdap:delta(A0,B0,p,Tau,w))          Tau = min((Tau-epsilon4,Tau,Tau+epsilon4),key=lambdap:delta(A0,B0,B,p,w))          epsilon1 *= .998          epsilon2 *= .998          epsilon3 *= .998          epsilon4 *= .998      returnA0,B0,B,Tau  # Initial parameters  A0_init = 68.5904  B0_init = 1697.85  B_init = 400.192  Tau_init = 8.325  INIT_PARAMS = (A0_init,B0_init,B_init,Tau_init)  defsigma(A0,B0,B,Tau):      return100*sqrt(sum(((val-f(A0,B0,B,Tau,t))/val)**2fort,valinenumerate(DATA,start=1))/120)  for w in range(1,21):      optimized = optimize(*INIT_PARAMS,w)      print(w,optimized,sigma(*optimized)) |
| --- |

**Table 2S**. Variation of the optimal values of (*A*_0_, *B*_0_, *B*, *τ*) with the statistical weight (*w*).

| *w* | *A*_0_ | *B*_0_ | *B* | *τ* / month | *σ*_rel_ (%) |
| --- | --- | --- | --- | --- | --- |
| 0 | 68.5904 | 1697.85 | 400.192 | 8.325 | 1.5333 |
| 1 | 68.43622627543299 | 1704.1396805440668 | 397.79782977223124 | 7.965225084712352 | 1.5281793095328158 |
| 2 | 68.48146981560056 | 1702.0778197669408 | 394.23636308081530 | 8.245092968334590 | 1.5234665795359033 |
| 3 | 68.51915777011526 | 1700.3712135736553 | 393.11146079194090 | 8.418846201392432 | 1.5205422715725385 |
| **4** | **68.55327541247976** | **1698.8323629244978** | **392.87004391394820** | **8.545746315091957** | **1.5182146417802076** |
| 5 | 68.58527247005439 | 1697.3926864285297 | 393.06160789715744 | 8.646998879255236 | 1.516186430905847 |
| 6 | 68.61582253085732 | 1696.0202158482907 | 393.4977830244699 | 8.732401064648977 | 1.5143528551744418 |
| 7 | 68.64529481591670 | 1694.6974231277557 | 394.08430743165576 | 8.807196516223385 | 1.5126681326783635 |
| 8 | 68.67390643632824 | 1693.4139922802601 | 394.76826719115735 | 8.874454489053180 | 1.511109163748705 |
| 9 | 68.70180064879729 | 1692.1631355853428 | 395.5176839805241 | 8.936113808523878 | 1.5096622965035067 |
| 10 | 68.72907486088417 | 1690.9402424622049 | 396.31205803637215 | 8.993458482894882 | 1.5083185684430318 |
| 11 | 68.75580123582402 | 1689.7419229916154 | 397.13769046958686 | 9.047381185598468 | 1.5070713372242044 |
| 12 | 68.78203368969479 | 1688.5656480131270 | 397.9851026945391 | 9.098520762105673 | 1.5059153563678558 |
| 13 | 68.80781452819431 | 1687.4094452126433 | 398.8475464518959 | 9.147349102195130 | 1.5048462017809299 |
| 14 | 68.83317776900638 | 1686.2717371959216 | 399.7201085957338 | 9.194223005050686 | 1.5038599934201538 |
| 15 | 68.85815213019312 | 1685.1512002261497 | 400.5991835581117 | 9.23941932366857 | 1.5029532141801838 |
| 16 | 68.88276153655767 | 1684.0467341709289 | 401.4820428664802 | 9.283156011067558 | 1.5021226493313735 |
| 17 | 68.90702541754132 | 1682.9574507578438 | 402.3665751120051 | 9.325605121124102 | 1.5013653466718240 |
| 18 | 68.93096326157051 | 1681.8824679978509 | 403.2512432229733 | 9.366911440459830 | 1.5006784656800207 |
| 19 | 68.95458951651295 | 1680.8211272362444 | 404.1347761057060 | 9.407188915846438 | 1.5000594129309077 |
| 20 | 68.97791952266130 | 1679.7727416501318 | 405.01624535845514 | 9.446537519214578 | 1.4995056721090174 |

The table displays the variation of optimal values (*A*_0_, *B*_0_, *B*, *τ*) with the statistical weight (*w*), along with additional parameters (*τ* / month and *σ_rel_* (%)). As you move down the table, you can observe how changes in the statistical weight (*w*) influence the optimal values of *A*_0_, *B*_0_, *B*, and *τ*, as well as the associated time constant and relative standard deviation. This kind of analysis is common in optimization studies where researchers seek the best-fitting parameters for a mathematical model that describes observed data.

***w* (Statistical Weight):** This column represents the statistical weight, which is a parameter used in the optimization process. It shows how changes in the statistical weight affect the optimal values. *A*_0_ is one of the optimized parameters. It represents a constant in the mathematical model or equation being used for the analysis. *B*_0_ is another optimized parameter. It represents a constant in the mathematical model or equation and contributes to determining the shape or characteristics of the model. ***B*:** *B* is an optimized parameter and, similar to *B*_0_, contributes to shaping the mathematical model or equation used for analysis. ***τ* / month (Time Constant per Month)** column represents the time constant per month, which is an important parameter in the context of the analysis. It indicates the time-related behavior or characteristic that is being modeled. ***σ_rel_*(%)** column represents the relative standard deviation, expressed as a percentage. It is a measure of the variability or dispersion of the data relative to the mean.

The adjustable parameters (*A*_0_, *B*_0_, *B* and *τ*) obtained by fitting the equations to the experimental values with a least-squares algorithm are given in Table 4, along with the relative standard deviation, *σ_rel_*, defined as follows:

$\sigma_{rel}= 100\times\sqrt{\frac{1}{N-m}\sum_{i=1}^{i=N} {(\frac{Y_{i,cal}-Y_{i,act}}{Y_{i,act}})}^{2}}$ (3S)

where *N* (=120) is the number of actual data, *m* (=4 in Eq. 11) is the number of parameters and *Y_i_* designates (*Cum_glob_* / *t*) at the time (*t*=*i*) in months, where the subscripts “*act*” and “*cal*” mean actual and calculated by Eq. 11, respectively.

**Figure 8S**. Variation of the optimal values of (*A*_0_, *B*_0_, *B*, *τ*) and relative standard deviation (*σ_rel_*) with the statistical weight (*w*).
